# Physician-assisted suicide: a review of the literature concerning practical and clinical implications for UK doctors

**DOI:** 10.1186/1471-2296-7-39

**Published:** 2006-06-22

**Authors:** Madelyn Hsiao-Rei Hicks

**Affiliations:** 1 Sections of Community (PRiSM) and Cultural Psychiatry, Department of Health Services Research, Institute of Psychiatry, King's College London, University of London, UK

## Abstract

**Background:**

A bill to legalize physician-assisted suicide in the UK recently made significant progress in the British House of Lords and will be reintroduced in the future. Until now there has been little discussion of the clinical implications of physician-assisted suicide for the UK. This paper describes problematical issues that became apparent from a review of the medical and psychiatric literature as to the potential effects of legalized physician-assisted suicide.

**Discussion:**

Most deaths by physician-assisted suicide are likely to occur for the illness of cancer and in the elderly. GPs will deal with most requests for assisted suicide. The UK is likely to have proportionately more PAS deaths than Oregon due to the bill's wider application to individuals with more severe physical disabilities. Evidence from other countries has shown that coercion and unconscious motivations on the part of patients and doctors in the form of transference and countertransference contribute to the misapplication of physician-assisted suicide. Depression influences requests for hastened death in terminally ill patients, but is often under-recognized or dismissed by doctors, some of whom proceed with assisted death anyway. Psychiatric evaluations, though helpful, do not solve these problems. Safeguards that are incorporated into physician-assisted suicide criteria probably decrease but do not prevent its misapplication.

**Summary:**

The UK is likely to face significant clinical problems arising from physician-assisted suicide if it is legalized. Terminally ill patients with mental illness, especially depression, are particularly vulnerable to the misapplication of physician-assisted suicide despite guidelines and safeguards.

## Background

A bill to legalize physician-assisted suicide in the UK recently progressed further than ever before in the British House of Lords. In 2005 Lord Joffe revised his Assisted Dying for the Terminally Ill Bill, originally written to legalize euthanasia and physician-assisted suicide (EAS), to narrow the focus to physician-assisted suicide (PAS) in England and Wales. The bill subsequently progressed to its second reading in the House of Lords in May 2006. Though peers voted to block the bill, Lord Joffe has declared his intention to continue reintroducing his bill to legalize PAS until it has proceeded through all parliamentary stages. Over the course of this legislative process, there has been little discussion of the clinical implications of physician-assisted suicide for the UK [1,2].

This paper summarizes some of the problematical clinical and practical issues that became apparent from a review I undertook to clarify what evidence exists to make a case for or against PAS. Searches were done on Medline and Google using the terms 'assisted suicide' and 'euthanasia'. Euthanasia was included because it overlaps with PAS in many respects and because its legalization has accompanied that of PAS in Belgium and in the Netherlands [3]. In addition, Lord Joffe has described this bill as the first step *"forward in incremental stages" (pp 53) *[4] and there is no reason to think that the goal to legalize euthanasia in the UK has been abandoned. Also reviewed were the House of Lords *Select Committee on the Assisted Dying for the Terminally Ill Bill Report and Evidence *[4], Lord Joffe's Assisted Dying for the Terminally Ill Bill [5], and the Oregon Department of Human Services report on PAS [6]. Websites of UK health professional societies were viewed for position statements.

In addition to drawing on the substantial medical and research literature on PAS, this paper addresses psychiatric issues that have been overlooked in the UK debate. The paper is framed in terms of the anticipated effects of PAS for UK doctors who would deal with these requests and addresses the following questions: What do doctors actually do in PAS? How will PAS likely affect a doctor's practice? How well do safeguards work to protect vulnerable patients and to prevent coercion, both overt and covert? And what role can doctors validly take in the UK debate on PAS?

## Discussion

### What does the doctor do in physician-assisted suicide?

Physician-assisted suicide is when a doctor provides a patient a lethal overdose of medication for self-administration with the explicit goal of enabling the patient to commit suicide. It is ethically and legally distinct from prescribing medication with the express goal of pain relief while understanding that death could occur earlier as a secondary effect (the double effect principle). GPs will deal with most assisted suicide requests in the UK, as they do in Oregon and the Netherlands.

In Oregon, doctors assist suicide by prescribing an overdose of barbiturates that the patient takes orally as several ounces of liquid. In 2005, the prescribing doctor was present at 23% of PAS deaths. The complication of vomiting occurred in 5% of cases. After taking the overdose, patients became unconscious in 2–15 minutes (median 5 minutes) and died within 5 minutes-9.5 hours (median 26 minutes). One patient took the overdose, lost consciousness in 25 minutes, and then regained consciousness 65 hours later. This individual did not obtain another PAS prescription and died 14 days later of the underlying illness [6].

In the Netherlands, doctors assisting suicide prescribe an antiemetic and an overdose of barbiturates in liquid or crushed tablet form. Euthanasia is by intravenous barbiturates followed by a muscle relaxant to paralyse breathing. Dutch doctors also administer high-dose opioids with the explicit intention of causing death. Despite acknowledging this to be euthanasia, Dutch researchers have not counted these cases in reported numbers of voluntary, involuntary, and unreported euthanasia [7] in their publications [8,9] or in evidence to the House of Lords [4]. Complications occur in 7% of assisted suicides. Doctors proceed to carry out euthanasia in 18% of initial PAS cases, usually because death took longer than expected, coma did not occur or the patient awoke from the coma [10].

Lord Joffe's bill would *"enable an adult who has capacity and who is suffering unbearably as a result of a terminal illness to receive medical assistance to die at his own considered and persistent request" *[5]. Similar to Oregon, the doctor who agrees to participate in the PAS process is responsible for determining the following: the patient has a terminal illness that will cause death within six months, the request is voluntary (uncoerced), he has mental capacity, and his 'unbearable suffering' (subjectively defined by the patient and either mental or physical) arises from the terminal illness, irregardless of whether suffering can be relieved or treated. The doctor ensures that a palliative care specialist sees the patient to provide information on palliative care. A referral to a second doctor is made to confirm these criteria.

### The likely scope of physician-assisted suicide in the UK

Most deaths by PAS are likely to occur for the illness of cancer and in the elderly. In 2005, 84% of Oregon PAS deaths were for cancer. Also in 2005, 68% of Oregon PAS deaths were of individuals aged 65 and older, consistent with previous years [6]. Oregon's PAS data have been cited somewhat out of context by some authors [1,11] who say that patients using PAS are 'younger', to imply that they are not elderly and so vulnerability is not an issue. To be more accurate, what the Oregon data show is that younger patients are more likely to use PAS than older patients if they receive a terminal diagnosis, but older patients constitute most PAS deaths. Also, the report states that the median age of patients dying by PAS is 70 as opposed to the median age of 78 in non-PAS deaths [6].

Oregon legalized PAS in 1997. Since a peak in 2003 of 13.6 PAS deaths/10,000 total deaths, the rate of PAS has stabilized with a 2005 rate of 12.0/10,000 deaths, which is about 1 in 800 Oregon deaths [6]. A recent survey comparing the UK to other European and Commonwealth nations shows that the illegal practice of euthanasia and PAS rarely occurs, with a current rate of 0% of deaths by PAS in the UK [12]. Although it has been estimated based on Oregon's experience that passage of Lord Joffe's bill would result in approximately 650 UK deaths by PAS annually [4], the UK is likely to have proportionately more PAS deaths than Oregon due to the bill's wider application to individuals with more severe physical disabilities. Lord Joffe's bill allows looser criteria for patients' signatures and a greater degree of physician assistance (Table 1) specifically to include individuals with greater physical disability in the PAS process.

**Table 1:** Some differences between physician-assisted suicide criteria in Oregon and in Lord Joffe's Bill

**What constitutes a 'signature' on the written request declaration**	
Oregon & Lord Joffe's Bill:	Signed by the patient.

Lord Joffe's Bill:	**OR **patient leaves his mark **OR **third party signs at patient's direction if patient cannot due to physical infirmity.

**Degree of assistance from the doctor in the suicide**	

Oregon & Lord Joffe's Bill:	Doctor prescribes overdose.

Lord Joffe's Bill:	**OR **Doctor prescribes overdose *and provides the means *for patient to self-administer if patient cannot take overdose orally.

In terms of how often individual doctors in the UK may face a PAS request, this will likely be infrequent. In 2005, 39 Oregon doctors wrote a total of 64 prescriptions for PAS overdoses, one writing eight [6]. Most Dutch GPs may do euthanasia or physician-assisted suicide (EAS) a few times in a lifetime, while a few EAS advocates may do up to eight a year [7]. The infrequency with which individual doctors are involved in PAS reflects two factors: First, most patients do not have a terminal illness, and the vast majority of those who do, do not request assisted suicide. Second, patients and families who want PAS tend to seek out doctors who will agree to their request, leading to some concentration of PAS in doctors who are known supporters of the practice. Patients are not required to forgo PAS if their long-term GP or other consulting doctors assess that they do not meet criteria. And some GPs may refer such patients due to concern over ethics, legal liability, or technical and clinical aspects of PAS. Though UK doctors may seldom be directly involved, all would need to be educated on PAS due to its clinical, legal and ethical complexity and seriousness.

### Coercion in physician-assisted suicide

The British Geriatrics Society believes that elderly patients are particularly vulnerable to coercion in PAS requests [13]. The problem of coercion may be exacerbated by the Joffe Bill's 'signature' criterion in which third parties can sign for physically disabled and dependent patients. But coercion potentially affects any PAS request and can do so at any stage of the process. It can affect the decision to request the overdose, whether the overdose is taken, and how long the patient chooses to live between making the request and overdosing. Coercion can be difficult for doctors to detect, and even when detected it is sometimes ignored despite guidelines (Table 2). The prevalence of coercion in EAS requests is unknown since it is unstudied and relatively few case histories [14-17] are described. But before assuming that the examples of Table 2 are unusual, it is useful to consider that Cases 2 and 4 were in the public domain because pro-EAS societies suggested them for newspaper and television documentaries, respectively, implying that these cases are considered paragons of the appropriate application of EAS.

**Table 2:** Cases of coercion in physician-assisted suicide and euthanasia

**Case 1, Oregon: **An 85-year-old cancer patient with worsening dementia requests PAS but her psychiatrist believes that she is being pressured by family. Nevertheless, she is then approved for PAS by a psychologist and receives assisted suicide [16].
**Case 2, Oregon: **Louise, who has a degenerative neurological disease, requests PAS. As her disease progresses, those in her network who support her suicide become increasingly anxious that she will become too mentally or physically incapacitated to act on her request. This includes her doctor, her mother, a friend who will be present at her suicide, and the Oregon Compassion in Dying PAS advocate who has arranged for a New York Times reporter to fly in and cover the suicide. Louise says she is almost ready but not quite. She wants a week to relax and be with her mother. On learning indirectly that her doctor thinks she will not be able to act if she waits, she appears startled. Her mother tells her, "It's OK to be afraid." She replies: "I'm not afraid. I just feel as if everyone is ganging up on me, pressuring me. I just want some time" [15].

**Case 3, The Netherlands: **A wife who no longer wishes to care for her sick, elderly husband gives him a choice between euthanasia and admission to a nursing home. Afraid of being left to the mercy of strangers in an unfamiliar place, he chooses euthanasia. His doctor ends his life despite being aware that the request was coerced [14].

**Case 4, The Netherlands: **Cees requests euthanasia one month after being diagnosed with ALS (MND). As required, his request is assessed by the primary doctor who will carry out the euthanasia and by a consultant. During their assessments, both doctors allow Cees's apparently resentful wife to answer all the questions directed to him, even though his speech is still understandable and he can type on a computer. His ambivalence about euthanasia is expressed by repeatedly pushing the date back. It is also expressed by weeping in response to the doctor's pro forma question of whether Cees is sure he wants to go ahead with euthanasia. His wife quickly answers affirmatively for him and then tells the doctor to move away from Cees, saying it is better to let him cry alone. At no point does a doctor ask to talk with Cees alone before his euthanasia [15].

### Transference and countertransference in physician-assisted suicide

Reasons that patients and doctors give for PAS such as 'dignity', 'autonomy' and 'control', have been extensively documented. In this section, I attempt to balance the wide recognition given to these reasons with an overview of some less recognized forces that can affect PAS decision-making: transference and countertransference. Transference and countertransference feelings are normal and can occur in any doctor-patient relationship. When these feelings heighten around emotionally intense issues, they can exert coercive pressure on clinical decision-making with an obligatory quality that is difficult to resist. Recognition is complicated by the frequent involvement of unacceptable feelings and urges that both doctor and patient wish to deny. That specialized training is needed to systematically recognize transference-countertransference may underlie the finding that Dutch GPs are worse than Dutch psychiatrists at recognizing when transference or countertransference has affected a request for EAS [18]. A survey of Dutch psychiatrists found that transference and countertransference influenced doctor-patient decision-making in 25% of all EAS requests for which psychiatric consultation was sought. Transference and countertransference influenced 19% of cases in which the request for PAS or euthanasia was granted, despite the advice of the consultant [18]. This study demonstrates the importance of transference and countertransference as potentially distorting forces in PAS requests but does not describe the prevalence of specific forms of transference and countertransference. This section outlines potential forms of transference and countertransference that can influence decision-making even in apparently patient-generated PAS requests [19] and provides examples from cases of PAS and euthanasia.

Transference is when a patient relates to the doctor in a way that primarily replicates other important, usually parental, relationships. It frequently acts on an unconscious level to covertly affect the patient-doctor interaction. As a general example, patients may relate to the doctor as an omnipotent parental authority figure. Their communications and behaviour may express a wish for approval, a wish for comfort and restoration, fear of abandonment, or rage at perceived abandonment [20]. In any suicidal patient, including the terminally ill, the request to die can be a plea for help or an attempt to be given a reason to live. A request for PAS can be an entreaty for the doctor to take the terminally ill patient's situation or despair more seriously, or a test of the doctor's true feelings about the patient's value now that he is nearing death [19,20]. One patient's request for euthanasia was described as *"the patient's way of 'testing' the medical team...to make sure they would not be abandoned. Moreover, as the patient had a difficult relationship with their family – who had asked for euthanasia to be carried out – this request enabled the patient to hear that they still had a certain value in the eyes of the medical team" (pp 592) *[21]. Another example is that of Mr. C., a 72-year-old man with severe obstructive lung disease. This patient asked his doctor, *"Can't you do something to just bring it to an end? ...Just put me out of my misery. It would save everyone a lot of trouble." His doctor replied rather awkwardly, "Even though you feel like a burden, I can't do that." Mr. C. asks, "Why not? You'd do it for your dog." His doctor answers, "Because you aren't a dog, Mr. C. You're my patient and I'm your doctor, and I'm trying to help you. And I'll keep trying to help you as long as I have to." Mr. C. took the doctor's hand in both of his and said, "Thank God. I thought everyone had given up on me" (pp 67–68) *[17]. The authors suggest that the doctor was powerfully influential at this vulnerable point in the patient's life, related to the transference of feelings about early caretakers onto the doctor and the doctor's role as caretaker. They propose that if the doctor had taken a neutral role or agreed, there would have been a different outcome. Certainly not all patients who request PAS are looking for reassurance of their value, but careful exploration of the patient's motivations, needs and wishes is clearly critical in order to avoid readily confirming a patient's diminished sense of worth and for a real understanding of what is at stake for a patient who requests PAS.

For some patients, the PAS request can be a paradoxical attempt to regain control over life by setting the conditions for death [19,20,22]. Patients with amyotrophic lateral sclerosis (ALS, also known as motor neuron disease or MND in the UK) have an increased perception of control over their disease management if they choose to hasten death [23]. The intense drive and motivation that some patients have for PAS can overwhelm doctors with a sense of urgency, momentum, and a sense of losing control. These very determined patients can have a powerful effect on doctors, and potentially on doctors' decisions: *"I've always sort of felt like patients should be in charge, but he was in charge of a process that I wasn't that familiar with" (pp 455) *[24]. *"And I remember thinking that it's kind of like a lot of things, you get your nose into the tent, and then the next thing you know, here you are in the middle of this thing, and it kind of takes on a mind of it's own" (pp 456) *[24]. *"It was like talking to a locomotive. It was like talking to Superman when he's going after a train" (pp 196) *[25]. *"In these cases it's just "boom" a patient walks in the door and "boom" you are right up there on this incredible interaction. It's very powerful emotionally" (pp 456) *[24]. As described in the last quote, the effect of being swept up is not necessarily unpleasant for doctors, but some doctors regretted that they ended up involved in the logistics of PAS before fully understanding the reasons for the patient's wish to commit suicide [24].

Some medically ill patients can experience a split or fragmented sense of self in which the 'sick' or 'bad' part of the self is felt to be alien, with the feeling 'I don't know the person I've become' or 'This isn't me' [20]. One man in a study of EAS attitudes in patients with HIV or AIDS described dying slowly, *"a little piece at a time" (pp 363) *[26]. In such cases, a request to die may express the fantasy of killing off the bad self, leaving the healthy self to survive or be reborn [20]. PAS requests may express rage: rage at the doctor for distancing himself or for not providing a cure, rage at the world, or rage at themselves. Rage can induce a wish for revenge with fantasies of harming others by dying [19,20]. EAS requests based on revenge have been documented in palliative care patients [21]. Guilt may motivate PAS requests in patients who equate their illness with personal failure. For example, it has been suggested that a patient might attribute her cancer to unacceptable emotions or bad deeds and a PAS request could reflect the need for self-punishment and atonement [19,20]. In one case, a teacher with end-stage AIDS is allowed through his doctor's exploration of his suicide wish to reveal how exhausted he is by trying to *"be a role model" and "put on a good face" for others (pp 216–217) *[27]. It would seem that he may feel guilty or ashamed at not living up to the perceived standards of others, and suicide allows him to save face. The author's [27] description of how the doctor's social intervention facilitated the patient and his loved ones adapting to his changed needs is an excellent example of how doctors might deal constructively with a terminally ill patient's wish for suicide. Not every request for PAS is determined by psychodynamics, but this cannot be known unless the doctor has the training and capacity to consider these possibilities and to talk with the patient in depth. To act on a PAS request because the patient is judged rational and competent without exploring the possible hidden meanings of the request is simplistic and can ignore the patient's unspoken needs [20].

Countertransference, the mirror image of transference, is when a doctor reacts to the patient based on his or her own intrapsychic issues or background. It is generally assumed that doctors can accurately assess patients' requests for PAS apart from their own intrapsychic conflicts and personal history. However, it has been cautioned that countertransference can affect doctors' assessments of PAS requests, potentially leading to errors in classifying patients as appropriate or inappropriate for assisted suicide [19]. Doctors who are affected by countertransference or who have psychologically committed themselves to PAS may be prone to accepting patients' reasons for PAS at face value without thorough exploration [28,29], as illustrated by the following examples. An Oregon doctor who provides PAS said, *"... I learned it very quickly... trying to talk somebody out of it or to really assess their motivation... the patients were quite resentful of that. Pretty quickly I could take at face value what they're saying and then spend most of the rest of the time exploring with them how they understand about their options" (pp 458) *[24]. Patients and their family members described doctors who were willing to prescribe a lethal overdose but who avoided discussing the patient's motivations or state of mind in requesting PAS. A wife said, *"My husband, with the advice of a doctor friend that lives in [another state], went to his cardiologist...And he told the doctor that he needed Seconal. And this doctor has known my husband for a long time, and all he said was, 'I trust you have a good reason', and gave it to him, a prescription for it" (pp 1261) *[30]. In a more extreme example, a family member found that after a visit with the patient's oncologist, the prescription for a lethal overdose had been tucked into her bag secretly during the appointment [30]. A case report of a man with terminal lung cancer is notable for the difference between assessments of his psychiatric history by clinicians who prioritized PAS and by clinicians who prioritized treatment (Table 3) [16].

**Table 3:** Clinical and legal complexity in a request for physician-assisted suicide

Mr. A, a 63-year-old Oregonian, called a PAS helpline to request PAS the day he received a diagnosis of terminal lung cancer. He was distraught and saw no purpose in chemotherapy, saying "I might as well end it". The worker informed him that he had actually called a group offering support and palliative care for individuals considering PAS. He ended up discussing his concerns and described having suicidal feelings that first began after his mother committed suicide when he was 21. After her death, he attempted suicide 3 times and was treated for depression in a psychiatric hospital. With the worker's support, he decided to get treatment for his depression from his GP and to start chemotherapy and radiation treatment which alleviated his physical symptoms. He disclosed his diagnosis to his daughter, who became involved.
One year later, he obtained a PAS prescription from a doctor who had actively publicized assisting the suicide of a depressed patient, and who did not consider a psychiatric consultation to be "necessary" for Mr. A. Another doctor associated with the PAS movement was in contact with Mr. A to discuss the PAS option. When asked if this doctor knew of his psychiatric history, Mr. A replied that they "didn't get into that. Our conversations were superficial".

Six months later, Mr. A was admitted involuntarily to a psychiatric ward for suicidal and homicidal thoughts. In addition to being diagnosed with a depressive disorder and narcissistic personality traits, he was diagnosed with intermittent delirium, probably from his numerous sedative medications. During his hospitalization, some of his doctors did not provide comprehensive psychiatric assessment or treatment, seeming to compartmentalize his suicidal symptoms under the rubric of PAS. Prior to his discharge, although firearms were removed from his house based on his risk to self and others, and guardianship was set up due to his "periods of confusion and impaired judgement", his treating doctors did not remove the assisted suicide drugs prescribed by the PAS doctor because they were unsure of their legal right to do so. Mr. A had now long outlived his original prognosis of 6 months, so a PAS doctor gave him a new prognosis of 6 months to live so that his assisted suicide would be "legal".

Mr. A's suicidal urge receded, but returned 3 weeks before his death when he experienced pain from constipation and from stopping his pain medicine in the midst of confusion and paranoia. He was desperate from the pain and on the verge of taking the overdose. His PAS doctor offered to sit with him while he took it. His GP and palliative care worker offered him reassurance, rehydration and a morphine pump. He accepted these interventions and his confusion, pain, fear and suicidality quickly cleared. He was much relieved in the remaining weeks of his life despite his physical deterioration [16].

One of the commonest countertransference reactions is over-identification with the patient. Doctors treating terminally ill patients can experience 'pseudoempathy', feeling the patient's suicidal wish is 'normal' and that they would feel the same way [20,29]. Doctors often over-identify with strong-willed, determined patients in PAS, as found in an Oregon study [24]. It is not always evident when 'identification' becomes 'over-identification'. For example, doctors working with HIV patients in the San Francisco Bay area were more likely to have assisted a suicide if they identified their sexual orientation as gay, lesbian or bisexual [31]. The authors believe this finding could have two explanations: The doctor's sexual orientation may facilitate a therapeutic identification, allowing a better understanding of the patient's situation, or it may contribute to over-identification that distorts doctor-patient decision-making around the patient's PAS request.

Two psychiatrists [32,33] analyzed a case of PAS as reported by Dr. Timothy Quill, an American internal medicine physician who is a PAS proponent. Early in his advocacy for PAS, Quill published articles [34,35] and a book [36] about assisting the suicide of a patient, Diane. Diane requested PAS soon after being diagnosed with leukaemia, with a 1 in 4 chance of survival with treatment. She told Quill how she was enraged at the oncologist's assumption that she wanted treatment and she was convinced that she would die during treatment. Diane asked Quill for PAS saying she wanted to be in control. Wesley [33] and Hendin [32] argue that Quill's over-identification with Diane and her need for control led to his rapid acceptance of her request for PAS, without exploring why she wished so desperately for control. Quill's over-identification can be seen in one of his articles about this patient when he asserts, *"Diane was a friend as well as a patient" (pp1039) *[35]. The titles of Quill's publications on Diane's case reinforce the impression that he values PAS as an expression of control and autonomy: 'Death and dignity: a case of individualized decision making' [34] and 'Death and Dignity: Making Choices and Taking Charge' [36]. Wesley [33] interprets that Quill's assumption that Diane's request *"made perfect sense" (pp 693) *[34], and his quick response to Diane's PAS request with a referral to the Hemlock Society, may have conveyed the message that he agreed *"if you cannot be fully independent, you are better off dead" (pp 483) *[33]. Quill's familiarity with the Hemlock Society, which was known to few doctors at the time, is proposed to imply that Quill had a pre-existing interest in PAS that may have influenced how he dealt with this patient's request [33]. Certainly Wesley's point is a fair one: *"It is not a neutral act to refer a patient contemplating suicide to the Hemlock Society..." (pp 483) *[33]. Similarly, it has been suggested that doctors may influence patients' decisions when doctors explicitly suggest PAS, as is common in the Netherlands [14].

As with many patients who request PAS, doctors can have a low tolerance for situations they cannot control. It is especially difficult to endure a terminally ill patient's suffering and to bear his distress [29,37,38]. In a survey, 44% of doctors admitted that they found the anxiety of a terminally ill patient sometimes unbearable [39]. Lack of knowledge about what to do may lead doctors to 'do something' by assisting suicide. The less knowledge doctors have of palliative care, the more they support EAS [40,41]. Once doctors know how to alleviate suffering in terminal cases and no longer feel helpless, support for PAS in those cases recedes [14]. Oncologists have described providing terminal care and the associated frustration and sense of failure due to limited therapeutic success as major factors in job burnout [42]. Hendin [14] suggests that some doctors, unaware of palliative care alternatives and unable to tolerate lack of control, may attempt to regain their illusion of mastery over disease and to alleviate their sense of helplessness or failure by taking an active role in the PAS process, making death a medical decision. Rene Diekstra, a pioneer of PAS in the Netherlands, described how some doctors coming before a committee that reviewed PAS cases were prematurely ready to provide PAS when feelings of helplessness about the patient's condition influenced them to overestimate the rationality or inevitability of the patient's suicide [32]. Fear of inadequacy and of abandoning patients by denying the PAS request can be observed in Dobscha et al.'s [24] interviews with Oregon doctors. One doctor favorable to PAS said, *"...I think I would just feel really uncomfortable if I couldn't help" (pp 455) *[24]. Whether or not a doctor chooses to provide PAS, the patient's request for PAS can be perceived as a rejection or a condemnation of the doctor's inadequacy. As one doctor said, *"It's almost as if your treatments and attempts to make the patient comfortable have been a complete failure if they're going to ask for that" (pp 455) *[24]. And another: *"I feel like there's something with physician assisted suicide, personally, where I see it as a rejection of care...somehow the patient is saying, 'Whatever you're doing isn't good enough. It's not meeting my needs."' (pp 455) *[24].

Some doctors feel deep disgust towards disease and can have a profound fear of death and the helplessness that accompanies illness. Dr. Lewis Thomas writes, in an unusually naked portrayal of these feelings, *"Death is shocking, dismaying, even terrifying...A dying patient is a kind of freak...an offense against nature itself" *[43]. Some individuals become doctors as a way of dealing with their death anxiety [44,45]. Doctors' fears of death and of other issues around PAS can contribute to their avoiding much-needed discussions with patients about their impending death, both in doctors who support and who reject PAS. An Oregon doctor said about a PAS request, *"I kind of dealt with the medical issues and I didn't square up with it...I avoided it" (pp 454) *[24]. This reaction can lead to doctors giving PAS prescriptions to patients without adequate evaluation [30], as described earlier. But importantly, this can also lead doctors who are against PAS to reject any discussion about death with the patient; in some cases the doctor severs the relationship altogether in reaction to a PAS request [46]. For example, a patient terminally ill with cancer *"described how she could detect that her oncologist became "really uncomfortable" talking about PAS or "anything" about dying, and she changed the subject for him. She said, "I learned that he's a baseball fan and much more comfortable if I change the topic to baseball...It's awful when you have to try to make them feel comfortable, but that's the way it is" (pp 1260) *[30]. In the case of another patient, who wept with frustration as she described trying to get her doctor *"to sit down and listen", "Her clinician's unwillingness to discuss PAS resulted in missed opportunities to connect with this patient's deepest concerns, which included her quality of life, her prognosis, and her suffering" (pp 1261) *[30].

Back et al. [30] observed that terminally ill patients used talking about PAS as a gateway to talking about dying and that a therapeutic relationship was often more important to patients interested in PAS than a lethal prescription. Whether or not doctors provided PAS, therapeutic relationships could be maintained based on open communication and on the doctor setting clear boundaries for his or her role [30], and, it would seem, on the doctor keeping the focus on the patient's feelings and needs rather than on the doctor's feelings about PAS. Meier et al. [47] give another example of how doctors who choose not to provide PAS can shut down their patients' attempts to discuss their impending death *: "Mrs T, a 55-year-old successful lawyer, had struggled with progressive renal cancer for several years and was increasingly distressed by her progressive dependency and feelings of isolation. She asked her doctor for advice on ending her life, saying that she "just [couldn't] take it any more." Her doctor recalls feeling distressed by her request and her evident despair and ill equipped to explore the reasons for it with her. Instead, she tried to encourage her, saying that she didn't believe in helping her patients die and that now was not the time to give up hope. "You are a fighter and I know that you want to beat this." She closed the visit by saying, "Hang in there," and then gave the patient a pat on the back. Mrs T...took an overdose of sleeping pills 1 week later" (pp 3011) *[47]. The authors suggest that the doctor's discomfort with both her patient's desperation and the request for PAS interfered with exploring the patient's reasons for the request, and may have left the patient feeling alone and without options.

Doctors can experience PAS very positively. Many in an Oregon study emphasized how involvement in PAS caused them to become better at talking about death and providing palliative care [24]. That the PAS request functions as the means towards having an open discussion about the patient's death echoes reports by patients and families who seek PAS [30]. Both studies [24,30] give the impression that for doctors who participate in PAS, PAS is the necessary stimulus to deal with death in a way that is meaningful and adequate for patients, and the doctors thereafter associate provision of PAS with the gratifying experiences of mastering death and of achieving a special relationship with their patients. The studies also give the impression that doctors who do not provide PAS often continue to avoid talking about death altogether. This pattern raises the question of whether most doctors do a disservice to patients if a PAS request is considered the necessary basis for addressing patients' needs around their death.

As another way of positively experiencing PAS, one doctor described the pleasure of embracing death while controlling its process so that it is done 'right' through PAS: *"You know, it's an odd thing, and you've probably heard this from other physicians, but if you don't sidestep the process of dying, it becomes almost as pleasurable a thing helping people die right as giving birth...And done well, the process of helping somebody die right is very rewarding" (pp 458) *[24]. This doctor's interesting equation of death with birth, two biological opposites, hints at an avoidance of the finality of death through using the fantasy of death as a 'rebirth'. The fantasy of reunion after death is another way that doctors and patients can deny the finality of death, as described in cases of PAS [32,33]. In a Dutch case that was highly publicized because PAS was used for psychological distress without any physical illness, a bereaved mother demanded PAS from a psychoanalytically trained psychiatrist, Dr. Chabot, saying that she felt *"pulled to her boys" (pp 146) *[32]. One son had died by suicide and the other by cancer. As 'Netty' drank her overdose of barbiturates during her PAS, she lay on her younger dead son's bed in his room with photographs of both dead sons next to her. Dr. Chabot suggested to her, *"Think of your boys" (pp 149) *[32] as she lost consciousness. It seemed that Dr. Chabot had colluded with his patient's fantasy of reunion after death [32]. In another case [32,33], Diane, whom Dr. Quill had provided with a prescription for a lethal dose of barbiturates, came to say goodbye before her suicide, promising him *"a reunion in the future at her favourite spot on the edge of Lake Geneva, with dragons shining in the sunset" (pp 693) *[34]. In the conclusion of his account of her PAS and of his participation, Quill writes that he wonders if he *"will see Diane again, on the shore of Lake Geneva at sunset, with dragons swimming on the horizon" (pp 694) *[34].

Some doctors share with their patient, or experience unilaterally, an intense sense of closeness and intimacy through PAS, as Hendin [32] proposes that Dr. Quill and Dr. Chabot did while assisting in their patients' suicides. A Dutch palliative care physician, Dr. Cohen, who estimated that he had performed euthanasia somewhere between 50 to 100 times and had consulted in many more, was asked why he became so involved with euthanasia. He replied, *"There is satisfaction in being involved in the terminal phase of life. You become part of a family...There is a special warmth and intimacy and harmony" (pp 137–138) *[32]. Dr. Cohen knew that some might find it strange, but he would often bring a bouquet of flowers to the euthanasia appointment [32]. A doctor's need for closeness can sometimes be intrusive to the patient or family. In the opinion of one family member, their PAS doctor *"lacked boundaries" (pp 1262) *[30]. This doctor had an intense relationship with the patient that included daily telephone calls and home visits. On the night the patient attempted PAS, the doctor carried out a backup plan after the oral overdose failed. The family member recounts that after the patient's death, *" [The physician] would go over to the hospital to see a patient, and she'd call me at 10 o'clock PM and say she wanted to come over [to our house] and sit in the room where he died and 'hang out.' And I'd say no, and she'd come over anyway" (pp 1262–1263) *[30]. After a couple of these incidents, the relative wrote to the doctor requesting no more contact because he felt burdened by her behaviour [30].

An assumption underlying the proposal that doctors, and only doctors, be legally sanctioned to assist suicide is that doctors are beneficent. Undercutting this assumption is another long-documented form of countertransference experienced by doctors: hatred of the patient, often expressed in the guise of love [29,48]. The effect of hostile countertransference on doctors' decision-making has been described in regard to patients in general [48] and with suicidal patients specifically [49]. Hostile countertransference can potentially affect doctors who accede to PAS requests and doctors who refuse them. I outline the theoretical origins and effects of hostile countertransference for PAS here, and follow this with an examination of possible hostile countertransference in two cases of PAS.

A doctor may resent the drain of terminally ill and suicidal patients on his time and emotions. He may unconsciously act out caregivers' aggressive feelings or distress, which can stem from exhaustion, their own over-identification with the patient, or hostility [19,29]. On a deeper level, a doctors' inability to cure can threaten his or her fantasy of omnipotence and provoke a sense of inadequacy, and this can trigger rage or hatred toward the patient [19,29,50]. Doctors may deal with countertransference hostility in a number of ways: They may attempt to disavow or counteract their hostility by becoming over-involved. They may feel obligated to accede to the patient's request for PAS. Doctors who disagree with PAS may abandon the patient, both therapeutically and emotionally. Doctors may transgress professional boundaries normally maintained. They may distance themselves and ignore opportunities to reassure the patient's fears or to explore his ambivalence about death and suicide. Some may simply act out their hostility by contributing to the patient's suicide [19,29,50]. For doctors in general, the understandable need to deny aggressive urges makes it particularly difficult for them to recognize this reaction to patients: *"In some way we are always reassuring ourselves that our motives are beyond question because we have chosen to spend our days in the business of understanding others and helping them to improve their lives" (pp 253) *[50].

During my search for examples of countertransference, I found no published analyses of hostile countertransference affecting EAS decisions. This could be due to a self-reporting bias against disclosing a taboo feeling, possible low participation of doctors with hostile countertransference in studies of doctors' characteristics in EAS, or perhaps because hostile countertransference is rare in the situation of EAS. I did find two accounts by doctors of their participation in PAS that suggest possible elements of hostile countertransference.

In his paper, 'The ambiguity of clinical intentions' [35], Dr. Timothy Quill explores his motivations in assisting the suicide of his patient. He asks, *"What were some of my true intentions in prescribing barbiturates for Diane?" *In his exact words, number 3 on his list of 'intentions' is this: *"3. To cause her death (to kill her). Diane was a friend as well as a patient. I wanted her to be able to live as long as she could find any meaning in her life, and then to die as peacefully as possible. I had no desire to determine the time or to be the agent of her death, and to say that I intended to kill her is outrageous. Yet, why the barbiturates? There are much safer drugs for sleep. My intentions must have been more complex" (pp 1039–1040) *[35].

In the above passage, Dr. Quill demonstrates his ambivalence about facing the nature of, as he later puts it, his *"multilayered intentions" (pp 1040) *[35]. He first states that he intended *"to kill her"*, then immediately disavows this intention with the disclaimer, *"to say that I intended to kill her is outrageous"*. His prescription, which was specifically given for and calculated to be a lethal overdose, is contemplated as something *"to cause death (to kill her)"*, then *"for sleep"*, then acknowledged as something that is 'not safe for sleep' *(pp 1039–1040) *[35]. What Quill does not write is interesting as well. He does not write that it was 'as though' or 'as if' he intended to kill her. He specifically writes that he 'intended' to kill her. Another intention he identifies is, *"5. To allow her to kill herself" (pp 1040) *[35]. But his intention, *"3. To cause her death (to kill her)" *is distinct from this. It stands alone and comes higher on his list. At no point in his paper does Quill write that he feels hatred or rage towards Diane or towards what she may have symbolized to him. But to intentionally kill someone is surely one of the most hostile acts possible, and from his own account it appears that assisting Diane's suicide had, on some level and to some degree, this meaning for Dr. Quill. As he summarizes, *"...multilayered intentions are present in most, if not all, end-of-life decisions" (pp 1040) *[35].

Dr. Jack Kevorkian is a pathologist who carried out a confrontational and exhibitionistic crusade for PAS in the United States for a decade until his sentence for second-degree murder in 1999 for assisting the suicide of a man with ALS (MND), an event broadcast on national television. His style and methods in providing PAS to a series of patients were so repugnant and bizarre that, with few exceptions [51], those on both sides of the PAS debate share an almost visceral aversion to referring to him in discussions of PAS. Certainly he is not representative of the average doctor who provides PAS. However, examination of his case is informative in terms of possible manifestations of countertransference in PAS. The following dialogue is quoted from a verbatim transcript of the videotape Dr. Kevorkian made of his meeting with Marjorie Wantz, Sherry Miller and their families to plan the assisted suicides of the two women the next day [52]. The videotape was made with their knowledge and broadcast over television. Marjorie Wantz was a 58-year-old woman with chronic pelvic pain and Sherry Miller was a 43-year-old woman with progressive multiple sclerosis. An edited version of their dialogue is presented in Table 4 with phrases italicized for emphasis.

**Table 4:** Extracts from the transcript of Dr. Jack Kevorkian's videotape of his meeting with Marjorie Wantz and Sherry Miller to arrange their physician-assisted suicides [52]

Dr. Kevorkian begins the meeting by identifying each patient, friend and family member present. He systematically checks with each patient and family member on their wishes, feelings and understanding of the situation. He confirms for the record that the patients wrote to him, that they clearly understand assisted suicide means they will die, that this is what they definitely want and that their wish has been consistent. Each woman is very clear that PAS will result in her death and that she wants to die. The patients and their families recount their struggles with the illness, previous suicide attempts gone wrong, and how they came to ask Dr. Kevorkian for assisted suicide. The general sense from this part of the transcript is that the patients and families are definite about wanting assisted suicide and that they are deeply relieved and grateful that this doctor is going to provide it. Kevorkian repeatedly checks with each woman, "Are you afraid at all? Do you have any fears?"

At this point, there is an abrupt shift when Kevorkian changes the focus of the group by beginning, "I think there should be several options for people...The one option is to humanely, quickly and painlessly to have life ended, that's one option...The second option is for example, let's take Sherry's case. Now Sherry has got a good heart and good organs, except the central nervous system. And I ask patients – I do this routinely because it's just sort of a research project...The second option...which is donating organs. Now, Marge you probably could have that option too. How old are you?" Marge replies, "Fifty-eight." Kevorkian goes on, "Very close to the limit of donating organs, though. And...you have an infectious process, too. Isn't that infected? So you probably couldn't donate organs, but Sherry could." "...*Sherry's liver could save two babies*...And the third option would apply to you and Sherry...It's a prolonged process in which you're put to sleep under anaesthesia like a hospital operation, routine operation. And you just won't wake up...*Option three is to do an experiment and you would get choices there. See, I want to give the patient the maximum latitude in choosing value of life and death*. As it is now, none of us really has a maximum opportunity to choose our value of our lives, and our death. *Our death is really valueless, it's negative*." "...So, these are options that patients should have because that maximizes the self determination."

Marjorie interjects, "What I want to know is..." and asks him a series of intent questions to make sure she will get a very detailed autopsy to find out what a doctor has "done wrong" to her during her pelvic surgeries. Kevorkian quickly brings the conversation back: "Now Marge, what would you pick?" and outlines again the "three options" of assisted suicide only, organ donation or experiments. Marge seems slightly thrown off, "I never have given it a thought, and I'm trying to think – [Kevorkian cuts her off] "You will have to think about it and we'll get back to you later on this. Sherry, what would you pick of the two options?" Sherry says, "I just want out...Although I've never really given it any thought." Others in the group become interested in Kevorkian's organ donation idea and start to ask detailed questions. Specific body parts are discussed in terms of their viability for organ donation: heart, central nervous system, kidneys, lungs, liver, eyes and corneas.

In response to Sherry's and Marjorie's choice of "We just want it quick, you know", Kevorkian several times assures them, "No one judges you that you just want a quick one. No one judges you. I mean, it's just the choice of a person himself or herself." "...Sure, that's understandable. And beside that, some people would say, "*What do I owe society*? It's done nothing for me." "I don't blame them for that..." "I understand your situation, when it's your own life and your own death, *you don't feel an obligation to other people*. I can see your reasoning there. There's no judgment at all on my part." Sherry's friend, Karen, now derails Kevorkian: "I'd like to know what's going to happen to Sherry after it's over." And the rest of the meeting moves into planning how to handle the media coverage and the logistics of the suicides the next day.

In his meeting, Dr. Kevorkian moves from the beginnings of a well-structured examination of the goals and understanding of these two women and their families about their wish for PAS. These individuals seem to be deeply relieved and grateful that they have found a doctor to end their suffering. Then, in a disorienting shift, Kevorkian moves the group's focus onto what is clearly an essential part of his agenda in providing PAS: his interest in organ donation and human experimentation. I believe that in terms of countertransference, the real interest here is not in his bizarre preoccupation, but rather in how he relates to these two women and what role he designates them in the context of his preoccupation, of which PAS is part. Interestingly, he couches his proposition in a caricature of patient autonomy, offering *"choices"*, *"maximum latitude in choosing"*, *"options"*, and *"self determination"*. The most revealing phrases he uses seem to be these: *"Sherry's liver could save two babies." "Our death is really valueless, it's negative..." *When Sherry and Marjorie just want PAS, they 'just want to get out quick', he counters, *"Sure... some people would say, 'What do I owe society?"' "...you don't feel an obligation to other people. I can see your reasoning there. There's no judgment at all on my part" *(Table 4). It seems that to Kevorkian, these two women are 'valueless' and 'negative' in their death. Their only value is if they allow themselves to be disassembled into their useful parts, as one would recycle the salvageable parts of a wrecked car. He almost seems impatient, and resentful, that they cling to pieces of themselves that they can't use anyway. In Kevorkian's written description to accompany a painting he did that shows a man clawing at the sides of a chasm as he falls screaming into a black void, Kevorkian writes, *"This depicts how most human beings feel about dying...Most of us will do anything to thwart the inevitable victory of biological death...pleading wantonly and unashamedly, clutching any hope of salvation through medicine or prayer..." *[53]. My sense is that the main feeling that Kevorkian conveys through his relating to Marjorie and Sherry, and through his reaction to the human terror of death, is contempt. He may affirm his patients' lack of fear and their wish to 'just get out' instead of 'clutching on', but he seems more to resent that they are not willing to abrogate themselves completely and on his terms. Freud commented that the opposite of love is indifference rather than hate [54]. It seems as if his two patients are not real enough to Kevorkian for him to hate them personally. But what they symbolize to him, the situation of contending with helplessness and terror in the face of death, is perhaps something that he reacts to with a deep hostility. On hostility and contempt, Gabbard [48] suggests that contempt can provide a self preservative function. A doctor might regard a patient with contempt as a way of exaggerating the differences between themselves and their patient in order to preserve their own identity and separateness (pp. 89) [48]. Kevorkian's most real relationship may be with his own death anxiety rather than with his patients.

Medicine, a profession dedicated to helping others, can provide an ideal opportunity to conceal sadism [50]. It is not unusual for altruistic individuals to have chosen the field of medicine as a defense against their own aggression, that is, to keep from hurting others [44]. Patients are generally protected from doctors acting on urges to kill by the ethical and legal boundaries prohibiting killing, to the extent that this is considered a non-issue in medicine apart from rare, deviant cases. The legal sanctioning of doctors to assist patients in killing themselves may create a new, undesirable conduit for the expression of hostile countertransference. Because of the difficulty of recognizing transference and countertransference in general, many doctors will deny or be unaware when these forces play a role in PAS decisions and monitoring is inherently compromised. Of additional concern, it has been found that even when transference or countertransference is recognized as driving patients' requests for EAS, with psychiatrists involved in the assessment process, it is not unusual for the patient's request for EAS to be granted anyway [18].

### Unrecognized depression in terminal illness and physician-assisted suicide

The PAS criteria for Oregon, the Netherlands and Lord Joffe's bill assume that a doctor assessing mental capacity will recognize depression and appreciate its influence on a patient's request for assisted suicide. Those who rely on this safeguard to prevent the provision of PAS to clinically depressed, suicidal patients further assume that when depression is recognized, PAS will not proceed. The evidence suggests that these assumptions are ill-founded. After training on depression, UK GPs recognize depression in only 39% of all depressed patients attending their practices [55]. Specialists are not necessarily better. Oncologists recognize 33% of mild-to-moderate cases of depression and only 13% of severe depression cases in their cancer patients [56], and nurses similarly under-identify severe depression in this group [57,58]. Recognizing depression in suicidal patients can be complicated by the phenomenon that having decided on suicide, some individuals appear far from incompetent to make treatment decisions. Rather, they are calm and relaxed while laying out an eminently cogent case for why they should be hopeless and want suicide. As part of the negativity of depression, a patient with a terminal illness may also denigrate palliative measures and dismiss the possibility of relief, making suicide seem an even more 'obvious' choice [19]. Depression can also affect the patient's perception of the doctor: Depressed cancer patients are more likely than non-depressed cancer patients to have increased trust in doctors who mention euthanasia or PAS explicitly [59].

Detecting depression in patients requesting PAS is particularly difficult for several reasons. First, doctors providing PAS are less likely to know the patient well due to the doctor-shopping that can occur in the patient's search for PAS. In Oregon, the median duration of the doctor-patient relationship before death by assisted suicide is 8 weeks (range 0 to 678 weeks) [6]. Second, depression is harder to diagnose in patients with terminal illnesses [37]. Depression is significantly overlooked and under-treated in late-stage cancer [56,60] and is generally under-recognized in palliative care settings [61,62]. Third, in severely ill patients, even when low mood is recognized, doctors tend to consider it 'normal': an understandable reaction to the situation. This can lead to treatment not being initiated or advanced [19] or to administration of EAS anyway, particularly if the patient is elderly and has a serious physical illness [18,63]. There is reason to think that under-recognition of depression will be replicated in UK PAS situations: UK GPs have been found to consider late-life depression 'understandable' even in the absence of terminal illness and to have a strong nihilism that nothing can be done for these patients [64]. Furthermore, UK GPs report that their treatment of depression is limited by their skills and by lack of resources including referrals and time [64,65].

Beyond the problem of recognizing depression, the influence of depression on the wish to die in terminally ill patients can be underappreciated. In the debate about PAS, individual studies are sometimes cited to argue either that depression is associated with the desire for PAS, or that it is not, depending on the study and on the individual's stance on PAS. In order to clarify the relationship between depression and the wish for PAS, I searched and found a total of 23 studies of terminally ill patients that examined both depression and the wish for a hastened death, including PAS or euthanasia, and that used a control group for comparison. These studies include the top three diseases for which patients die by PAS rather than by their illness: ALS (MND), HIV/AIDS, and cancer [6]. The pattern that emerged was this: Fifteen of the 18 studies that used a standardized measure of depression, whereby the patient is asked a series of questions specifically to identify a depressive illness, found that depression is significantly associated with requests for PAS, euthanasia, or the wish for a hastened death (83% of studies, 95% CI 61 to 94) [23,59,66-81]. In contrast, none of the four studies that relied solely or predominantly on a clinician's or caregiver's impression of whether the patient had depression, sometimes based on recall going back four years [82], found any significant association between depression and requests for PAS, euthanasia, or the wish for a hastened death (0% of studies, 95% CI 0 to 0.5) [26,82-84]. One study that used a standardized assessment had mixed results, finding that depression was associated with greater desire for a hastened death, but not with whether the patient would consider asking for a PAS prescription [85]. Reliance on clinicians' superficial impression of depression is of questionable validity in studies examining the relationship between depression and the wish for hastened death since it has been found repeatedly that clinicians under-recognize depression in medically ill patients, as described above. In conclusion, studies that systematically assess depression in terminally ill individuals do not show that all requests for PAS arise from depression, but they do provide robust evidence that depression plays a role in the desire for a hastened death, including PAS or euthanasia, in a significant proportion of those with terminal illness.

A description by Dutch researchers of their pre-study hypothesis in regard to the relationship between depression and requests for euthanasia illustrates both the difficulty of recognizing depression in terminally ill patients and the clinical bias that can affect assessments of depression in the context of EAS: *"Our clinical impression was that such requests were well-considered decisions, thoroughly discussed with healthcare workers and family. We thought the patients requesting euthanasia were more accepting their impending death and we therefore expected them to be less depressed. To our surprise, we found that a depressed mood was associated with more requests" (pp 6611) *[80]. Using standardized depression questionnaires, the authors found that depressed cancer patients were four times more likely to request euthanasia [80]. It is concerning that examination of their data shows euthanasia proceeded on an unspecified number of these patients with identified depression, without any indication of depression treatment or reassessment of the euthanasia request.

As distinguished from depression, hopelessness is another factor clearly associated with the wish for a hastened death, PAS and euthanasia [23,66-68,71,75,81,86], and can have a stronger effect than depression [71,86]. Pain has not been found to play a significant role in the wish for a hastened death [59,67,68,71,74,75,78], with the exception of two studies [69,70]. In HIV/AIDS patients, cognitive impairment has been associated with a wish for hastened death [87]. It is not only the case that mental illness or distress can affect requests for PAS. In turn, requests for PAS can affect the quality of treatment of mental illness and distress: Patients with mental illness who then develop a terminal disease can receive substandard treatment for their psychiatric relapses after requesting PAS, partly because doctors become confused by the competing interests of PAS and clinical care [16] (Table 3).

### Decisions about physician-assisted suicide and euthanasia can change

Requests for EAS are often motivated by terror of what will happen rather than by current symptoms [14,16,21]. Facing uncertainty, some patients fill the vacuum with fantasies and fears. When fears and palliative care needs are addressed, the request for an assisted death usually disappears, as illustrated by specific cases [16,32,46]. But a tendency to adhere to the minimum legal standards for assessing mental capacity for PAS, and for doctors to cut off discussion of PAS if they do not agree with the practice, can cause missed opportunities for resolving the unnecessary fears that can motivate PAS requests, for example, a patient's fear that she will be forced to have a feeding tube [28] or the unnecessary fear of a man with ALS (MND) that he will die in pain, with the sensation of burning legs, and choking on his own secretions [46].

In the terminally ill, the desire to die has been found to decrease over time [69]. Over two months, half of terminally ill cancer patients had changed their minds about wanting euthanasia or PAS. Decision instability was particularly associated with depressive symptoms [70]. Antidepressant treatment can alleviate the desire for death due to major depression even in terminally ill patients [88]. In addition to antidepressants, effective depression treatments in the terminally ill include rapid-acting psychostimulants, electroconvulsive therapy and focused psychotherapy [89-92]. Improvement in depression is generally accompanied by an increased desire for life-sustaining interventions in the elderly and the terminally ill [93-96]. In the Netherlands, pain alleviation resulted in 85% of patients withdrawing their requests for EAS [97]. In an Oregon study of PAS requests, 46% of patients who received substantive palliative interventions changed their minds about assisted suicide, as compared to 15% of patients who received no substantive intervention. Interventions had been implemented in about half of instances where they were recommended [98]. Delirium can affect mental capacity and decision stability in PAS [16]. Delirium can be present in up to 85% of patients in the final stages of cancer, yet is often misdiagnosed or unrecognized by non-psychiatric doctors [92]. In one study, patients' requests for euthanasia were more persistent when both the family and the patient wanted euthanasia, suggesting that when families reinforce the request it may be harder for patients to change their minds and retract their request [21].

### Psychiatric evaluations and physician-assisted suicide

In Oregon and in Lord Joffe's bill the patient is to be referred to a psychiatrist or psychologist if a PAS doctor believes that the patient's judgment is impaired by a psychiatric or psychological disorder. Referral rarely takes place. In Oregon, only 5% of PAS cases were referred for a psychiatric evaluation in 2005, down from 31% in 1998 [6]. In the Netherlands, EAS requests for reasons of physical illness are to be referred for a psychiatric opinion as per the Oregon guidelines. Furthermore, in all cases of EAS for a mental disorder, which became legal in the Netherlands in 1994, referral to a psychiatrist is advised. Ten percent of Dutch EAS deaths were for reason of a mental disorder. But only 4% of all Dutch EAS requests were referred to a psychiatrist, indicating a serious under-referral rate for the psychiatric assessment of EAS requests. What is more, EAS is sometimes carried out when a psychiatrist has assessed that the case does not meet criteria, despite Dutch guidelines [18].

The application of EAS to patients solely for a mental illness is worth addressing here as an aside since Lord Joffe has stated that he favors an eventual *"much wider application" (pp53) *[4] and that the current bill *"is based on the principle of autonomy and only a competent patient can make a decision in relation to his or her own life. For people who are mentally incompetent there needs to be, perhaps, a different system..." (pp56) *[4]. Euthanasia and PAS are legally applied to individuals solely for their mental disorder in both Belgium and the Netherlands [3]. Patients requesting EAS for a mental disorder in the Netherlands tend to be younger and female, with psychiatric diagnoses in completed EAS cases consisting of mood disorders, personality disorders, and 'unspecified' [18]. Highly publicized Dutch cases include EAS by psychiatrists of a woman for major depression and bereavement [7] and a woman for depression and anorexia nervosa [99]. Patients in the Netherlands and in Belgium who want EAS for reason of their mental illness generally ask their psychiatrists to assess their request for assisted suicide or euthanasia. If the psychiatrist agrees, he usually takes on the additional role of prescribing the lethal overdose or carrying out euthanasia [3]. It has been observed that evaluating a patient's wish to die while treating the patient for a mental illness destroys the therapeutic boundaries that facilitate treatment [29,99], and the situation of a psychiatrist carrying out euthanasia on his patient is suggested to be *"a highly complex situation, which has to be treated cautiously" (pp 406) *[3].

For patients requesting PAS for their terminal physical illness, a thorough psychiatric evaluation can be a crucial catalyst for treatment and can alleviate distress [14]. But the evidence suggests that mandating psychiatric evaluations for all PAS requests will not solve the problem of patients inappropriately receiving PAS. Only 6% of Oregon psychiatrists were very confident that in one evaluation they could adequately assess whether a patient had the mental capacity for a PAS request [100]. Indeed, it has been suggested that the assessment of PAS requests in the terminally ill is so complex that it should be done by liaison psychiatrists [37]. But because many UK hospitals to not have access to a liaison psychiatry service [4], UK patients will not have uniform access to psychiatrists qualified to assess their PAS requests. Bias may exist in psychiatric competency evaluations because the majority of psychiatrists willing to evaluate a patient's capacity for PAS are in favour of PAS [100] and psychiatrists' ethical views for and against PAS affect clinical opinions regarding patient capacity for PAS in different directions [101]. In addition, psychiatrists are not immune to the effects of transference and countertransference, as illustrated by the earlier case example of Dr. Chabot's participation in his patient's assisted suicide [32].

Cases of PAS in Oregon show that if a patient is assessed as depressed or lacking capacity for PAS, the patient or her caregivers may continue to seek assessments until the desired outcome is provided by agreeable doctors, including psychiatrists and psychologists [16,28]. In the Australian Northern Territory, where EAS was legal for a short period before revocation and psychiatric assessments for EAS were mandatory, psychiatrists found that patients requesting EAS sometimes withheld key information because they saw the psychiatric assessment as a legal hurdle to be overcome. A gatekeeper role for psychiatrists was found to be unworkable [102].

### The effect of physician-assisted suicide on doctors

Studies have varied in how they represent the effect of EAS on doctors, possibly because of doctors' mixed feelings and the varied effects of participating in PAS. In a national survey in the US, 58% of doctors who had assisted a suicide reported feeling very comfortable with having taken that role, yet only 39% would definitely do it again [103]. Among oncologists, 53% reported receiving comfort from helping a patient with EAS and 24% regretted having performed EAS. A third felt that the emotional burden associated with performing EAS had affected their practice of medicine, some positively by making them more sympathetic listeners and others negatively, for example through emotional burnout [104]. Dealing with requests for assisted suicide or euthanasia is emotionally intense and often engenders discomfort [24,32,105,106]. A Dutch doctor who had carried out euthanasia many times said, *"The price of any dubious act is doubt...I don't sleep for the week after" (pp 138)*, and *"The idea that each case gets easier and easier is just rubbish" (pp 137) *[32]. Doctors rarely seek support from their colleagues when dealing with PAS requests, and contend with the issues in professional isolation [24,106]. In doctors who control their aggression through the practice of helping others [44], or whose sense of self is carefully constructed on the basis on being a beneficent and caring figure [29], participating in PAS may create a sense of instability or conflict. For example, one doctor said, *"I find I can't turn off my feelings at work as easily...because it does go against what I wanted to do as a physician..." (pp 457) *[24]. Another said that ending a patient's life made him or her feel *"conflicted, at odds with myself [and my role]" (pp 510) *[104]. And another worried about *"playing God a little too much" (pp 510) *[104]. Dobscha et al. [24] suggested that some Oregon doctors feared being 'damaged' by participating in PAS, giving the example of a doctor asking, *"Are you doing something that you're going to be uncomfortable with later?...you've got an indelible mark upon your soul?" (pp 453–454) *[24]. Oregon doctors usually feel unprepared to deal with PAS requests, having dealt with few in their practice, and worry about lack of training and making legal mistakes. Not knowing the patient well makes decision-making harder. Most who choose to assist in suicide find that it requires a large investment of their emotions and time. Doctors whether they had refused or acceded to the PAS request did not regret their decision, yet many who had acceded were uncertain whether they would do so again [24]. One described the surprise of experiencing the difference between abstract agreement with PAS and carrying out PAS: *"Yeah, there is a huge gap between idealistic agreement with a thought and actually being involved in it. It's just an immeasurable gap that I hadn't anticipated" (pp 454) *[24]. Others described an unsettling mismatch between the reality and what they had imagined as the type of person they would assist to commit suicide: *"I kind of imagined that if I had been asked it would be by somebody that was already at the state of being in pain and feeling like they were a burden. And at that point, she was living on her own and doing very well" (pp 454) *[24]. *"But to actually do this to a person who is functional, who is not clearly terminally ill, that is a, that's a whole different ballgame" (pp 454) *[24]. Doctors also need to consider how they would feel if a patient they assisted in suicide were found not to have a terminal illness. The Royal College of Pathologists points out that post-mortem studies consistently show a 30% error rate in diagnosing cause of death [4]. In a recent case, a woman with depression falsified documents to create a history of terminal liver cirrhosis. The Swiss doctor who was thus deceived and administered her lethal injection committed suicide after discovering from a routine autopsy that she had no physical disease [107].

### Guidelines and reporting in physician-assisted suicide

As shown by the examples above, countries that legalize PAS or euthanasia using safeguards such as in Lord Joffe's bill can decrease but not eliminate the number of patients who are inappropriately assisted in suicide despite not meeting PAS criteria. In regard to the success of guidelines in achieving full reporting of PAS and euthanasia, this varies substantially. The Oregon Department of Human Services notes that it can monitor PAS only in patients who legally receive prescriptions for lethal medications and not in patients and doctors who may act outside the provisions of the law [6]. However, a survey of dying Oregonians identified no unreported cases of PAS [108], suggesting either no, or negligible, underreporting of PAS in Oregon.

In contrast, reporting of PAS and euthanasia in the Netherlands is very poor. Despite multiple revisions of the Dutch EAS monitoring system, almost half of EAS cases were still unreported by doctors in 2001 [109]. In cases of unreported EAS [110], the most common reasons given by doctors for not reporting EAS were primarily self-serving: 92% of GPs did not report 'to spare himself the bother of a judicial inquiry' and 79% of nursing home doctors did not report because they believe that the law has no place in EAS decisions. This suggests that the power of legal safeguards to determine the practice and monitoring of PAS may be limited primarily by doctors' willingness to comply based on their own interests.

### The medical profession and physician-assisted suicide

Lord Joffe's bill would make doctors uniquely exempt from the legal offence of assisting suicide, an offence that would still apply to all other categories of person with a penalty of up to 14 years' imprisonment. In a culture that encourages medical action, therapeutic omnipotence, and technological aspects of care [29], the administration of death by doctors on request may be culturally attractive. In common with other Western cultures, British society's ideal of 'the good death' increasingly incorporates the value of personal autonomy in terms of control over the detailed circumstances of death, and also idealizes the nature of the process by which doctor and patient arrive at the conclusion that PAS is the best means to achieve that end [22]. In view of Switzerland's proof that the technical aspects of assisting suicide do not require a doctor's skill, it would seem that despite PAS proponents' emphasis on personal autonomy, doctors are needed on some deeper level to fulfill another sort of role in PAS. It may be analogous to the situation of transference; British society wishes doctors to be omnipotent and beneficent, providing reassurance, sanctioning its choices, and absolving it of responsibility for life and death decisions.

### UK doctors should consider their stand in the physician-assisted suicide debate

In a recent representative survey of UK GPs and hospital specialists who reported on their most recently attended patient death, 97.4% did not think that a new UK law allowing euthanasia or PAS would have enabled the patient to receive better or more appropriate care, and it was rare for doctors to feel that the current law had interfered with their preferred management of the patient's death [12]. Medical professional societies in the UK have varied in their response to Lord Joffe's bill to legalize PAS. The British Geriatrics Society opposes PAS and considers elderly patients to be vulnerable to its misuse [13]. The Royal College of Psychiatrists considers assisting suicide to be incompatible with the psychiatrist's role of trying to prevent suicide, and is deeply worried about the possible pressures that legalized PAS could place on psychiatric patients [111]. Both the Royal College of General Practitioners and the Royal College of Physicians reversed from neutrality to opposition to the legalization of PAS. The British Medical Association is currently taking a neutral stance and considers the legalization of physician-assisted suicide to be 'a matter for society'. Like many others, the BMA has responded to concern about PAS by emphasizing the need to 'press for robust safeguards'. But as described in this paper, safeguards have not adequately protected patients in other countries from the misapplication of PAS and doctors should not feel reassured that they will in the UK. Findings from other countries also indicate that patients who experience depression during their terminal physical illness may constitute a group particularly vulnerable to the misapplication of PAS, especially if they are elderly or have a previous history of mental illness.

The British government is taking a neutral stance while observing the reactions of society, Parliament and UK professionals to Lord Joffe's bill. UK doctors have access to an adequate body of research and clinical expertise to inform themselves, government and the public of the clinical repercussions of legalizing PAS in the UK. It is incumbent on doctors to formulate their position on PAS and to decide whether providing PAS to the few who would correctly qualify in legal terms truly outweighs our responsibility to advocate for the protection of other patients who would be place at risk by its legalization.

## Summary

• A bill to legalize physician-assisted suicide (PAS) in the UK recently made significant progress in Parliament and will be reintroduced in the future.

• Requests for PAS and doctors' decisions to assist suicide can be influenced by coercion and by unconscious motivations in doctors, patients and caregivers.

• Depression is greatly under-recognized in terminally ill patients and increases the risk of the inappropriate use of PAS.

• Evidence from other countries shows that safeguards do not adequately protect vulnerable patients from the misapplication of PAS.

## Competing interests

An early draft of this paper was provided to the Law Commission for its review of the murder law in England and Wales as it pertains to suicide pacts and mercy killing and to the Royal College of Psychiatrists in response to a call for members' comments on the Assisted Dying for the Terminally Ill Bill. Subsequently, the author was asked to join a working group developing the Royal College of Psychiatrists' statement on physician-assisted suicide. An early draft of the paper was provided in response to a request by Professor the Baroness Finlay of Llandaff, a member of the House of Lords Select Committee on the Assisted Dying for the Terminally Ill Bill. A limited number of summary points and examples from an earlier version of this paper was presented by the author to the All Party Parliamentary Group on Dying Well 'Assisted Dying for the Terminally Ill Bill' seminar at the House of Lords on 27 April, 2006.

## Pre-publication history

The pre-publication history for this paper can be accessed here:


